# Supplementary material for: Content-rich biological network constructed by mining PubMed abstracts
Source: BMC Bioinformatics. 2004 Oct 8;5:147. doi: 10.1186/1471-2105-5-147 (PMC528731; doi:10.1186/1471-2105-5-147)
Supplement: Additional File 2 — The original results of the above study (non-essential files are deleted to keep the file size under the limit set by BMC bioinformatics). [file 1471-2105-5-147-S2.bz2 › chilibotAdditionalFile2/dip10/435ID7622448E10380/html/CCL5_CCR3.html]

 


 **CCL5** and **CCR3** 
  
Found 149 abstracts in PubMed, retrieved 10.  
 

 What does Google say? 
 PDF only 
| .edu only 

---

**Interactive relationship** (e.g. stimulation, inhibition, etc)

**Non-interactive relationship** (e.g. studied together, co-existance, homology, etc.)

- RESULTS Mast cells, activated eosinophils, T cells, IL 4 cells, IL 5 cells, eotaxin cells, RANTES  [ **CCL5** ]  cells and  **CCR3**  cells were all increased in the lesions of KD as compared with those in normal tissue.  Ref: 12653173 Clin Exp Allergy, 2002
- The sources of IL 4, IL 5, eotaxin and RANTES  [ **CCL5** ]  and the expression of  **CCR3**  were examined by immunostaining of serial sections with antibodies to IL 4, IL 5, eotaxin, RANTES  [ **CCL5** ]  and  **CCR3** , and antibodies to tryptase, ECP EG2 and CD3.  Ref: 12653173 Clin Exp Allergy, 2002
- METHODS We examined the number of tryptase cells, EG2 cells, CD3 cells, IL 4 cells, IL 5 cells, eotaxin cells, RANTES  [ **CCL5** ]  cells and  **CCR3**  cells in five specimens of KD versus normal tissues by immunohistochemistry.  Ref: 12653173 Clin Exp Allergy, 2002
- Despite there being no difference in expression of CC chemokine receptor 3  [ **CCR3** ] , the priming effect of RANTES  [ **CCL5** ]  and eotaxin was significantly enhanced in eosinophils from the patients.  Ref: 12797483 Eur Respir J, 2003
- Matching pairs of receptors ligands were found to be coordinately expressed, including TGFbetaRII with TGFbeta3, FGFRII and cysteine rich fibroblast growth factor receptor CRF 1 with FGF7 and FGF12, CCR1,  **CCR3** , CCR5 with RANTES  [ **CCL5** ]  and calcitronin receptor like gene CALCRL with adrenomedullin.  Ref: 12584245 J Cell Sci, 2003
